# Production of CFTR-ΔF508 Rabbits

**DOI:** 10.3389/fgene.2020.627666

**Published:** 2021-01-22

**Authors:** Dongshan Yang, Xiubin Liang, Brooke Pallas, Mark Hoenerhoff, Zhuoying Ren, Renzhi Han, Jifeng Zhang, Y. Eugene Chen, Jian-Ping Jin, Fei Sun, Jie Xu

**Affiliations:** ^1^Center for Advanced Models for Translational Sciences and Therapeutics, University of Michigan Medical Center, University of Michigan Medical School, Ann Arbor, MI, United States; ^2^Unit for Laboratory Animal Medicine, University of Michigan Medical School, Ann Arbor, MI, United States; ^3^In Vivo Animal Core, University of Michigan Medical School, Ann Arbor, MI, United States; ^4^Division of Cardiac Surgery, Department of Surgery, Davis Heart and Lung Research Institute, Biomedical Sciences Graduate Program, Biophysics Graduate Program, The Ohio State University Wexner Medical Center, Columbus, OH, United States; ^5^Wayne State University School of Medicine, Detroit, MI, United States

**Keywords:** CRISPR/Cas9, cystic fbrosis, CFTR-ΔF508, rabbits, gene edit

## Abstract

Cystic Fibrosis (CF) is a lethal autosomal recessive disease caused by mutations in the gene encoding the cystic fibrosis transmembrane conductance regulator (CFTR). The most common mutation is the deletion of phenylalanine residue at position 508 (ΔF508). Here we report the production of CFTR-ΔF508 rabbits by CRISPR/Cas9-mediated gene editing. After microinjection and embryo transfer, 77 kits were born, of which five carried the ΔF508 mutation. To confirm the germline transmission, one male ΔF508 founder was bred with two wild-type females and produced 16 F1 generation kits, of which six are heterozygous ΔF508/WT animals. Our work adds CFTR-ΔF508 rabbits to the toolbox of CF animal models for biomedical research.

## Introduction

The cystic fibrosis transmembrane conductance regulator (CFTR) is a cyclic adenosine monophosphate (cAMP)-dependent chloride (Cl) channel at the apical membranes of most epithelial cells. Loss of CFTR function causes cystic fibrosis (CF), a fatal autosomal recessive disorder with a disease frequency of 1 in 2,000 live births and a carrier rate of approximately 5% in the Caucasian population (Cutting, 2015).

More than 2,000 mutations on the CFTR gene have been identified,^1^ with the most common one being the deletion of phenylalanine residue at position 508 (ΔF508 or ΔF). Approximately 70% CF patients are ΔF508/ΔF508 homozygous. In addition, another 20% patients are of heterozygous compound mutations with ΔF508 on one allele and a different mutation on the other allele.

In 2019, the US Food and Drug Administration (FDA) approved Trikafta, marking a breakthrough in the CF drug development journey. Trikafta is a combination of three drugs: two CFTR correctors (VX-445 and VX-661) and a CFTR potentiator (VX-770). While Trifafta provides benefits to the majority of CF patients including those carrying one or two alleles of the dF508 mutation (Heijerman et al., 2019; Middleton et al., 2019), the consensus in the community is that CF is far from being cured and continued efforts should be dedicated to the development of novel therapeutics, for example gene editing mediated correction of CFTR mutations.

Clustered regularly interspaced short palindromic repeats/CRISPR Associated Protein 9 (CRISPR/Cas9) is originally discovered as a core member in the bacterial adaptive immune system (Price et al., 2016). It is now most known as the gene editing nuclease of choice (Khalil, 2020). In action, the CRISPR/Cas9 uses a guide RNA (gRNA) to locate the target sequence, where it efficiently generates double stranded breaks (DSBs), which are repaired by the error-prone non-homologous end joining (NHEJ) pathway or the homology directed repair (HDR) pathway. In recent years, CRISPR/Cas9 has become a mainstream tool in biomedical research. For example, it can be employed to generate gene knockout and knock-in animals as disease models. Our team has established a robust platform in generating knockout and knock-in rabbit models (Yang et al., 2014; Song et al., 2016; Yang et al., 2016; Song et al., 2017; Yang et al., 2019; Song et al., 2020). Furthermore, CRISPR/Cas9 can be used to correct disease causing mutations hence holds the promise for gene editing based therapeutics. In genetic diseases such as CF, the hope has been that CRISPR/Cas9 may enable a permanent correction of the intrinsic defect (i.e., the CFTR mutation).

Several groups including us have reported efficient gene editing of the CFTR gene in stem cells and in stem cell-derived organoids (Ruan et al., 2019; Geurts et al., 2020; Vaidyanathan et al., 2020). However, no one has reported successful gene editing therapy in a preclinical animal model system. Toward this goal, in the present work, we generated CFTR-ΔF508 rabbits by CRISPR/Cas9. These animals are useful not only as a model for the study of CF pathogenesis, but also for the development of gene editing strategies to correct the most prevalent ΔF508 mutation of CF.

## Materials and Methods

The animal maintenance, care and use procedures were reviewed and approved by the Institutional Animal Care and Use Committee (IACUC) of the University of Michigan, an AAALAC International accredited facility. All procedures were carried out in accordance with the approved guidelines.

### CRISPR/Cas9 Construction and sgRNA Synthesis

The Cas9 expression plasmid JDS246 was obtained from Addgene. Single guide RNAs (sgRNAs) were designed using the CRISPOR software (Concordet and Haeussler, 2018) and synthesized as chemically modified sgRNAs by Synthego (Menlo Park, CA, United States).

Cas9 mRNAs were transcribed *in vitro*, capped and polyadenylated using the T7 mScript^TM^ Standard mRNA Production System (C-MSC100625, CELLSCRIPT, Madison, WI, United States). Cas9 mRNA and sgRNA were diluted in RNase-free TE buffer (1 mM Tris–Cl pH 8.0, 0.1 mM EDTA), stored in −80°C in 10 μl aliquots, and were thawed and kept on ice before microinjection.

### Microinjection and Embryo Transfer

Sexually matured female New Zealand White (NZW) rabbits were superovulated by subcutaneous injection of follicle-stimulating hormone (FSH, Folltropin-V, Bioniche Life Sciences, Canada) twice/day with a dosage of 3 mg for the first two injections, 5 mg for the next two injections and 6 mg for the last two injections. Seventy-two hours after the first FSH injection, a single intravenously injection of 200 IU human chorionic gonadotropin (hCG, Chorulon, Intervet, Holland) was administered to induce ovulation. The superovulated females were mated with a male rabbit immediately after hCG injection. Sexually matured recipient female rabbits were synchronized by stimulate mechanically in the vagina and intravenous injection 200 IU hCG. Eighteen hours post insemination (psi), the superovulated rabbits were euthanized. The oviduct ampullae were recovered, flushed with 10 ml of Hepes buffered manipulation (HM) medium containing 25 mM TCM 199 (#12350039, Life Technologies, Grand Island, NY, United States) supplemented with 10% fetal bovine serum (FBS, #12003C, Sigma, St. Louis, MO, United States), and the recovered oocytes were observed under a microscope for the occurrence of fertilization, and then kept in the HM medium at 38.5°C in air.

Microinjection was performed on pronuclear stage embryos 19–21 h psi using a micromanipulator under an inverted microscope equipped with a differential interference contrast (DIC) device. Rabbit embryo was held with a holding glass pipette (120–150 μm diameter) in the HM medium. A mixture containing 150 ng/μl Cas9 mRNA and 50 ng/μl sgRNA, and 100 ng/μl donor oligo or plasmid DNA were used for cytoplasm microinjection. Injected embryos were washed three times in embryo culture medium, which consisted of Earle’s Balanced Salt Solution (E2888, Sigma) supplemented with non-essential amino acids (M7145, Sigma), essential amino acids (B-6766, Sigma), 1 mM L-glutamine (25030-081, Life Technologies), 0.4 mM sodium pyruvate (11360-070, Life Technologies), and 10% FBS. The injected embryos were surgically transferred into the oviducts of a synchronized recipient doe. Twenty to thirty embryos were transferred to one recipient doe. For *in vitro* validation, instead of transferring to a recipient doe, the injected embryos were washed and cultured *in vitro* for additional 3–4 days until they reach blastocyst stage.

### Confirmation of Gene Targeting Events

For *in vitro* validation of gRNAs, blastocyst stage embryos were pooled and lysed, genomic DNA extracted, and the whole genome was replicated using a REPLI-g^®^ Mini Kit (Qiagen, Germantown, MD, United States) following the manufacturer’s protocol with slight modification. Briefly, for harvesting denatured DNA, 3.5 μl Buffer D2 was added to the embryos, mixed by vortexing and centrifuged briefly. The samples were incubated on ice for 10 min. After that 3.5 μl Stop Solution was added, mixed by vortexing and centrifuged briefly. For replication, 2 μl of the denatured DNAs were added to 8 μl master mix and incubate at 30°C for 10–16 h. Then REPLI-g Mini DNA Polymerase was inactivated by heating at 65°C for 3 min. The PCR products were purified, and Sanger sequenced at the University of Michigan DNA Sequencing Core. The sequences were analyzed by using the online software ICE Analysis by Syntheco^2^ to determine the efficiencies of gRNAs, indicated by the rates of insertions and deletions (indels) at or close to the target locus (Supplementary Figure 1).

To determine the genotypes of animals, ear skin tissues were biopsied, and genomic DNA extracted. For animals produced in Condition (i) and (ii) where short length oligo donors were used, genomic DNAs were PCR amplified using primer set F/R (F: CCTCCAACCCTATCCCAACACTCTG; R: ATGATGGGCTAGGTTGGTGTATTAAA, Supplementary Figures 2, 3). For animals produced in Condition (iii) where a long length ds-donor was used, genomic DNAs were PCR amplified using primer sets LF/LR (LF: ACCATCT TTAATCATGTAGTTTCA; LR: AATCTTCCAATACCTTTCT GCTCATAA) and RF/RR (RF:GTTTCCAGACTTCGCTTC; RR: AATTTCCCAAACAACTACT) (Supplementary Figure 4). PCR products were purified, and Sanger sequenced at the University of Michigan DNA Sequencing Core. The sequences were analyzed by using the online software ICE Analysis by Syntheco.^3^ Animals carrying the desired knock-in sequences are considered knock-in animals.

### Off-Target Analyses

Potential off-target loci associated with sg02 in the rabbit genome were predicted by using an online off-target analysis tool CRISPOR (Concordet and Haeussler, 2018). Top 8 potential off-target loci that fall on an exon or an intro (Supplementary Table 1) were selected for off-target analysis, by using corresponding primer sets (Supplementary Table 2) to PCR amply the sequence, followed by T7EI (see below) assays to determine any indel events.

### T7EI Assay

The T7 endonuclease I (T7EI) assay was conducted as previously described (Xu et al., 2018). Briefly, the purified PCR products were denatured and re-annealed and digested with T7EI (M0302L, New England BioLabs, Ipswich, MA, United States) for 30 min at 37°C, and then run in an agarose gel. Non-perfectly matched DNA (presumably indel sites) would be recognized and cleaved by T7EI leading to two cleaved bands; whereas the perfectly matched DNA would not be recognized and cleaved by T7EI hence leading to only one band (the unedited band).

### Necropsy and Histology

Tissues were collected at necropsy following humane euthanasia, and immersion fixed in 10% neutral buffered formalin. Tissues were trimmed and processed through graded alcohols, cleared with xylene, and embedded in paraffin. Tissues were sectioned at 5 μm, mounted on microscope slides, and stained with hematoxylin and eosin (H&E) for microscopic analysis by a board-certified veterinary pathologist.

## Results

### Validation of Guide RNAs

**FIGURE 1:**
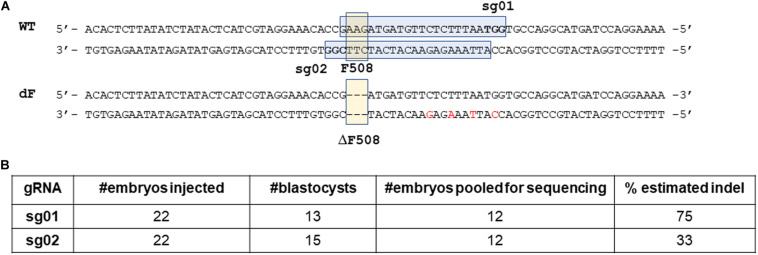
Design and validation of guide RNAs. **(A)** illustration of gRNA design. Blue box: sgRNA sequence. Bold letters within the blue box: PAM sequence. Yellow box: the F508 or the ΔF508 sequence. **(B)** *In vitro* validation results of sg01 and sg02.

We first analyzed the F508 proximal sequence of the rabbit CFTR gene (NCBI GeneID: 100009471) and designed two guide RNAs: sg01 and sg02 (Figure 1A).

We then microinjected sg01 or sg02 into pronuclear stage embryos to test their efficiencies *in vitro*. Similar blastocyst developmental rates were achieved in both groups (Figure 1B). To determine the rates of insertions or deletions (indels), we pooled 12 blastocytes from each group, extracted genomic DNAs, followed by PCR and Sanger sequencing. The sequencing results were analyzed by ICE online software,^3^ which estimated that the indel rate was 75% or 33% by sg01 or sg02, respectively (Figure 1B and Supplementary Figure 1).

Because both gRNAs passed our quality control threshold of 30% indel generating capacity, both were chosen for knock-in experiments.

### Production of ΔF508 Founder Rabbits

To knock-in the ΔF508 mutation, we designed two single stranded oligonucleotide (ssODN) donor templates (donor-oligo-01 and donor-oligo-02) and one double stranded donor template (ds-donor-01). Donor-oligo-01 is 130 nucleotides (nt) long and carries the ΔF508 mutation as well as 6 silent mutations (Supplementary Figure 2). Donor-oligo-02 is 120 nt long that carries the ΔF508 mutation but without any other mutations (Supplementary Figure 3). Ds-donor-01 is 3.9 kilobases (kb) long with 1.5 and 2.4 kb homology arms on each side, and carries the ΔF508 mutation and 4 silent mutations (Supplementary Figure 4).

We then used three conditions: Condition (i) sg01 + donor-oiligo-01; Condition (ii) sg02 + donor-oligo-02; or Condition (iii) sg02 + ds-donor-01, along with Cas9 encoding mRNA for embryo microinjection, followed by embryo transfer.

**FIGURE 2:**
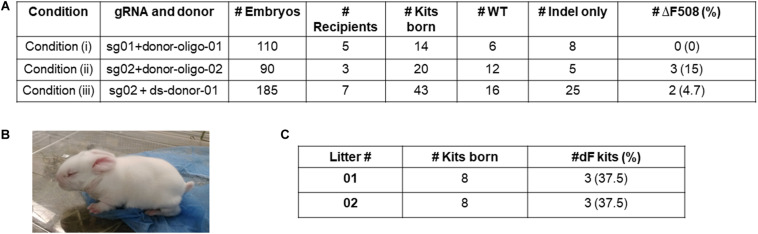
Production of ΔF508 founder rabbits. **(A)** Summary of embryo transfer and genotyping results. **(B)** One founder ΔF508 kit. **(C)** Summary of breeding outcome to generate F1 generation ΔF508 rabbits. #Indel only refer to number of animals that carry non-ΔF508 indel mutations. #ΔF508 refers to number of animals that carry ΔF508 allele.

For Condition (i), we transferred 110 embryos to five pseudopregnant recipients, and obtained 14 kits. Genotyping of the ear skin biopsy samples revealed that eight kits carried indel mutations, however none had the ΔF508 mutation (Figure 2A).

For Condition (ii), we transferred 90 embryos to three recipients, and obtained 20 kits, of which five possessed undesired indel alleles only, and three (15%) possessed the ΔF508 allele (Figure 2A).

For Condition (iii), we transferred 185 embryos to seven recipients, and obtained 43 kits, of which 25 possessed undesired indel alleles only, and two (4.7%) possessed the ΔF508 allele (Figure 2A).

Together, we produced multiple ΔF508 founder rabbits (exampled in Figure 2B) from Condition (ii) and Condition (iii) but none from Condition (i).

### Germline Transmission of the ΔF508 Allele to the F1 Generation Rabbits and Off-Target Analysis

We next worked to test the germline transmission capacity of the founder ΔF508 rabbits. One male animal from Condition (ii) was bred with two wild-type females. A total of 16 kits were born, and six were confirmed as heterozygous ΔF508/WT animals (Figure 2C and Supplementary Figure 5). These F1 generation ΔF508/WT animals look indistinguishable from their WT littermates.

One concern for CRISPR/Cas9 based gene editing is the potential off-target mutations. We therefore evaluated the top potential off-target mutations that fall on the exon or intron regions in one founder (#163) and five F1 generation ΔF508/WT animals (#254, 255, 263, 264, 265). In the selected top eight loci, no off-target mutations were detected (Supplementary Table 1 and Supplementary Figure 6), indicating that there are minimal off-target mutations in these dF508 animals.

### Production of a Compound Heterozygous ΔF508/KO Rabbit

**FIGURE 3:**
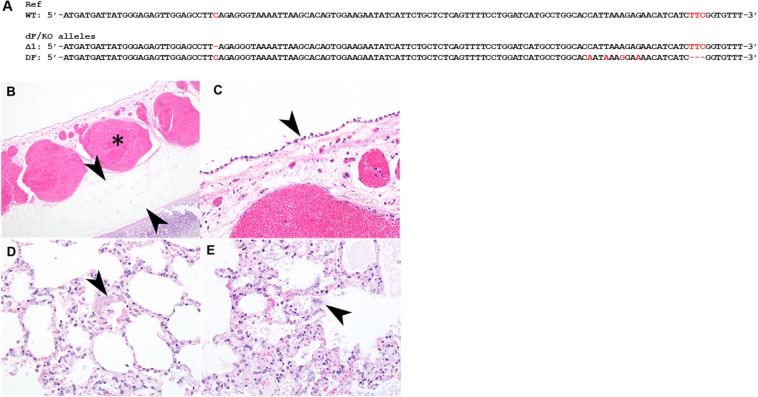
Histopathology of the tracheal and lungs of a heterozygous compound ΔF508/KO rabbit. **(A)** Illustration of allele sequence of the ΔF508/KO rabbit. Red letters indicate positions of mutations. **(B)** The submucosa of the trachea was thickened with moderate to marked amounts of edema (arrowheads) and submucosal blood vessels were markedly congested (asterisk). **(C)** tracheal epithelium was markedly flattened and attenuated (arrowhead). **(D)** Lung contained variable amounts of fibrin within alveoli (arrowhead), variable amounts of edema, and multifocal rod-shaped bacterial colonies [**(E)**, arrowhead].

Many CF patients carry heterozygous compound mutations. We previously produced CFTR knockout (CFTR-KO) rabbits and recently reported the phenotypes of these CFTR-KO rabbits (Xu et al., 2020). To test if heterozygous compound CF rabbits can be produced, we bred one male ΔF508 founder from Condition (iii) with a heterozygous CFTR knockout female rabbit and successfully produced a compound heterozygous ΔF508/KO rabbit. One allele of this animal has the ΔF508 allele while the other allele has the deletion of one nucleotide (Δ1) mutation (Figure 3A).

This ΔF508/KO CF rabbit survived 58 days, a lifespan that is similar to those of CFTR-KO rabbits (Xu et al., 2020). Postmortem examination revealed severe intestinal obstruction (Supplementary Figure 7). This is also similar to the observations in CFTR-KO rabbits, in which gut obstructions is the primary cause of mortality (Xu et al., 2020).

Formalin fixed samples of lung and trachea of this animal were subjected for histopathology. In H&E stained sections of trachea, the tracheal epithelium diffusely was attenuated and flattened, with scant cytoplasm (Figures 3B,C). There was moderate to marked congestion and edema within the underlying submucosa. In H&E stained sections of lung, there was mild to moderate edema within alveoli multifocally, alveoli contained variable amounts of fibrin, and there was loss of detail of alveolar walls (Figure 3D). There were increased numbers of heterophils within the vasculature or within alveolar capillaries. There were numerous rod-shaped bacteria multifocally (Figure 3E) within alveolar spaces, without associated inflammation.

These results demonstrate the feasibility of generating heterozygous compound ΔF508/KO rabbits, and suggest that ΔF508/KO rabbit may manifest typical CF phenotypes including intestinal obstruction and airway inflammation and infections.

## Discussion

In the present work, we produced CFTR-ΔF508 rabbits by using the CRISPR/Cas9 system. Importantly, one founder animal transmitted the ΔF508 mutant allele to its offspring, thereby satisfying the gold standard of transgenic animal production. Follow-up work is needed to establish the homozygous ΔF508 rabbits and comprehensive characterize their CF phenotypes.

Interestingly, although sg01 was shown to have higher indel generating capacity than sg02, all ΔF508 founder rabbits were produced from the groups that used sg02 but none from the sg01 group. We reason that the most possible explanation to the differential outcome between sg01 and sg02, as suggested by an early report (Paquet et al., 2016), is the distances between the PAM (protospacer adjacent motif) location and the targeted mutation site. Sg01’s PAM is further away from the F508 locus than that of sg02. Consequently, sg01’s cutting site is 13 base pairs (bps) from the F508 locus; whereas sg02’s cutting site is right at the F508 locus. This result underscores the importance of PAM location in Cas9 mediated knock-in applications.

The ΔF508 rabbits are a new addition to the CF mammalian animal model family, which currently consist of two rodent species, mouse (Grubb and Boucher, 1999) and rat (McCarron et al., 2020), and four non-rodent species: pig (Stoltz et al., 2015; Yan et al., 2015), ferret (Sun et al., 2010; Yan et al., 2015; Sun et al., 2019), sheep (Fan et al., 2018), and rabbits (Xu et al., 2020). CF mouse models were the first developed (Semaniakou et al., 2018). Different CF mice, including knockout, ΔF508, G551D and others, have made significant contributions toward our understanding of the disease and the development of therapies. However, unlike human patients, CF mice rarely show pulmonary pathophysiology nor obvious pancreatic pathology and liver problems. Similar to mice, CF rats, both knockout and ΔF508, develop gut obstructions but are otherwise normal in the pancreas, liver and lungs (Dreano et al., 2019). In the non-rodent models, CF ferrets (knockout and G551D), CF pigs (knockout and ΔF508) and CF sheep (knockout) were generated by nuclear transfer. CF ferrets and pigs, have been shown a closely mimicking pathology that is observed in CF patients, including lung, pancreatic and liver phenotypes that are not often found in CF mice. However, neither pig, ferret nor sheep is a convenient laboratory species, and they are associated with high maintenance cost and require special animal handling skills. Most recently, we reported the production of CFTR knockout rabbits that show many typical CF phenotypes (Xu et al., 2020). However, the specific knockout genotypes in these rabbits are not found in CF patients, hence not optimal for the development of mutation specific gene editing strategies.

The ΔF508 rabbits therefore may represent a useful model by offering several desirable features. Comparing to the non-rodent CF models (i.e., pigs, ferrets, and sheep), rabbit is a classic animal species that can be easily housed in most research facilities, and many experimental procedures are well established. Furthermore, rabbit has a short gestation time (30 days) and large litter size, making herd expansion very efficient. Comparing to CF rodent models (i.e., mice and rats), observations from the ΔF508/KO rabbit generated from the present work highly suggest that ΔF508 rabbits may be more clinically relevant, i.e., manifesting some typical CF phenotypes that rodent models failed to demonstrate, in line with our recent findings in the CFTR-KO rabbits (Xu et al., 2020). While this is only a single case, which needs follow-up studies in large number of homozygous ΔF508/ΔF508 rabbits to verify, the finding is exciting and promising.

One other advantage of the ΔF508 rabbit model over that of the mouse model is the relatively longer lifespan. A laboratory NZW rabbit can live beyond 6 years; whereas the lifespan of a mouse is 1–2 years. This is particularly important in the development of gene editing therapy strategies, as the longer lifespan allows a longer observation window for potential side effects, which do not always manifest in short term. For example, in the late 1990s, gene therapy for primary immunodeficiency was conducted using a gamma-retroviral vector. Strikingly, 5 of 20 patients developed leukemia 2–6 years after the gene therapy due to the integration of the vector in the vicinity of oncogenes (Hacein-Bey-Abina et al., 2003; Howe et al., 2008). Such long-term safety risks are beyond the lifespan of a rodent, but are legitimate considerations for gene editing therapies including those for CF. In this context, the ΔF508 rabbit provides a tool to monitor long-term efficacy and safety of novel therapies.

In summary, we successfully produced CFTR-ΔF508 rabbits by CRISPR/Cas9. These animals add a valuable tool to facilitate the study of CF pathogenesis and the development of novel therapies including gene editing therapeutics.

## Data Availability Statement

The raw data supporting the conclusions of this article will be made available by the authors, without undue reservation.

## Ethics Statement

The animal study was reviewed and approved by Institutional Animal Care and Use Committee (IACUC) of the University of Michigan.

## Author Contributions

YC, FS, and JX conceived the idea. JX, YC, J-PJ, and FS designed the experiments. DY, XL, BP, MH, JZ, ZR, RH, and JX conducted the experiments and analyzed the data. JX, YC, J-PJ, and FS wrote the manuscript. All authors contributed to the article and approved the submitted version.

## Conflict of Interest

The authors declare that the research was conducted in the absence of any commercial or financial relationships that could be construed as a potential conflict of interest.

## Footnotes

1 https://cftr2.org/

2 https://ice.synthego.com

3 https://ice.synthego.com
