# Supplementary material for: Production of CFTR-ΔF508 Rabbits
Source: Front Genet. 2021 Jan 22;11:627666. doi: 10.3389/fgene.2020.627666 (PMC7862758; doi:10.3389/fgene.2020.627666)
Supplement: Supplementary file 1 [file Table_1.DOCX]

**Supplementary Data**

**Title**

Production of CFTR-∆F508 rabbits

**Authors**

Dongshan Yang ^1,#^, Xiubin Liang ^1,#^, Brooke Pallas ^2^, Mark Hoenerhoff ^3^, Zhuoying Ren ^1^, Renzhi Han ^4^, Jifeng Zhang ^1^, Y. Eugene Chen ^1^, Jian-Ping Jin ^5^, Fei Sun ^5, &^, Jie Xu ^1, *^

**Supplementary Figures**

**Supplementary Figure 1.** Indel analysis output by the ICE online tool. (A) Indel results of embryos injected with sg01. (B) Indel results of embryos injected with sg02.


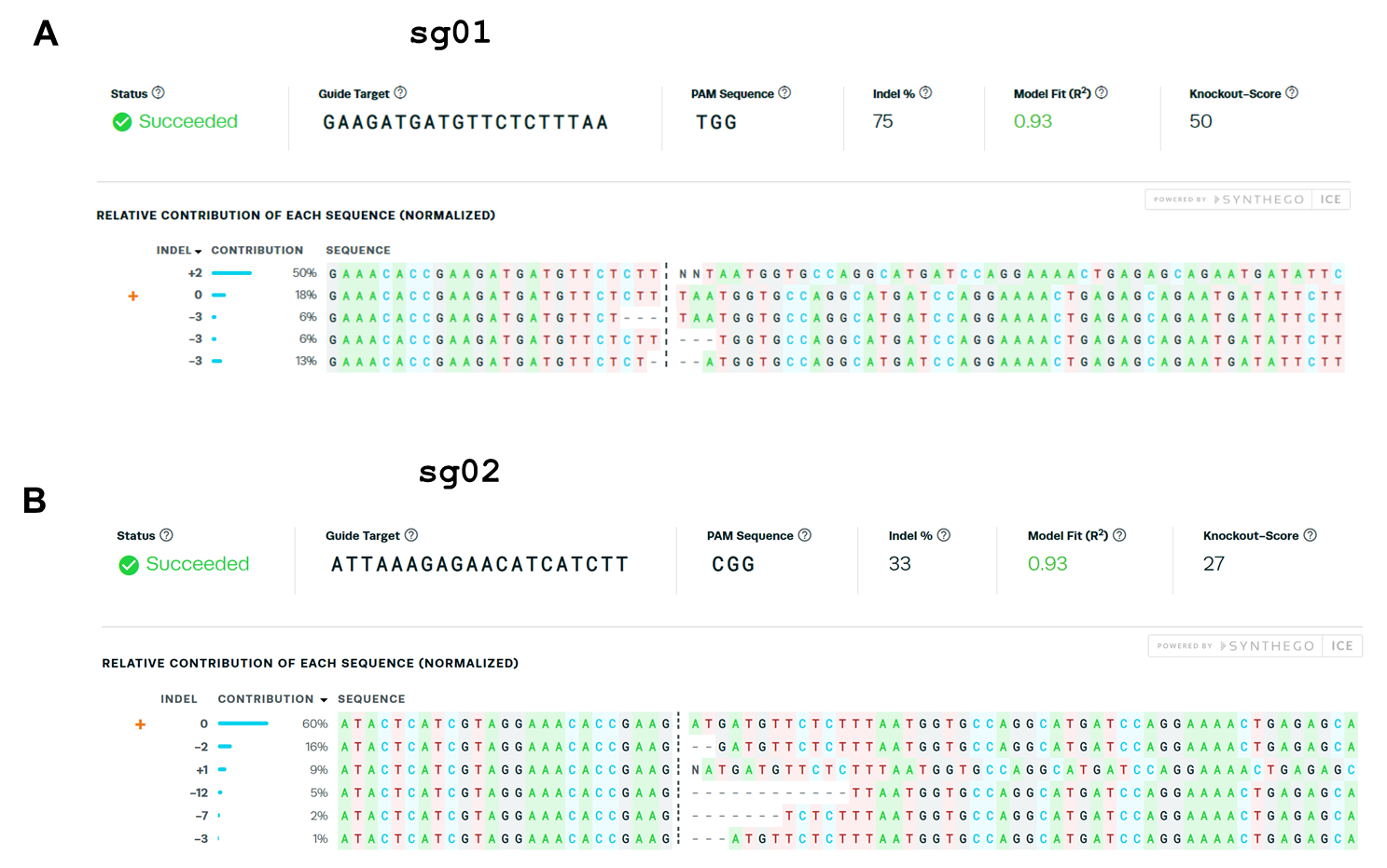


**Supplementary Figure 2.** Illustration of Condition (i). (A) Wild type (WT) allele sequence. Underlined sequences refer to the Forward primer sequence, Reverse primer sequence and the donor target sequence. Bold letters refer to the gRNA sequence. Red letters indicate the F508 locus. (B) Donor-oligo-01 sequence. Red letters indicate mutated nucleotides. Box: dF508 locus.

**
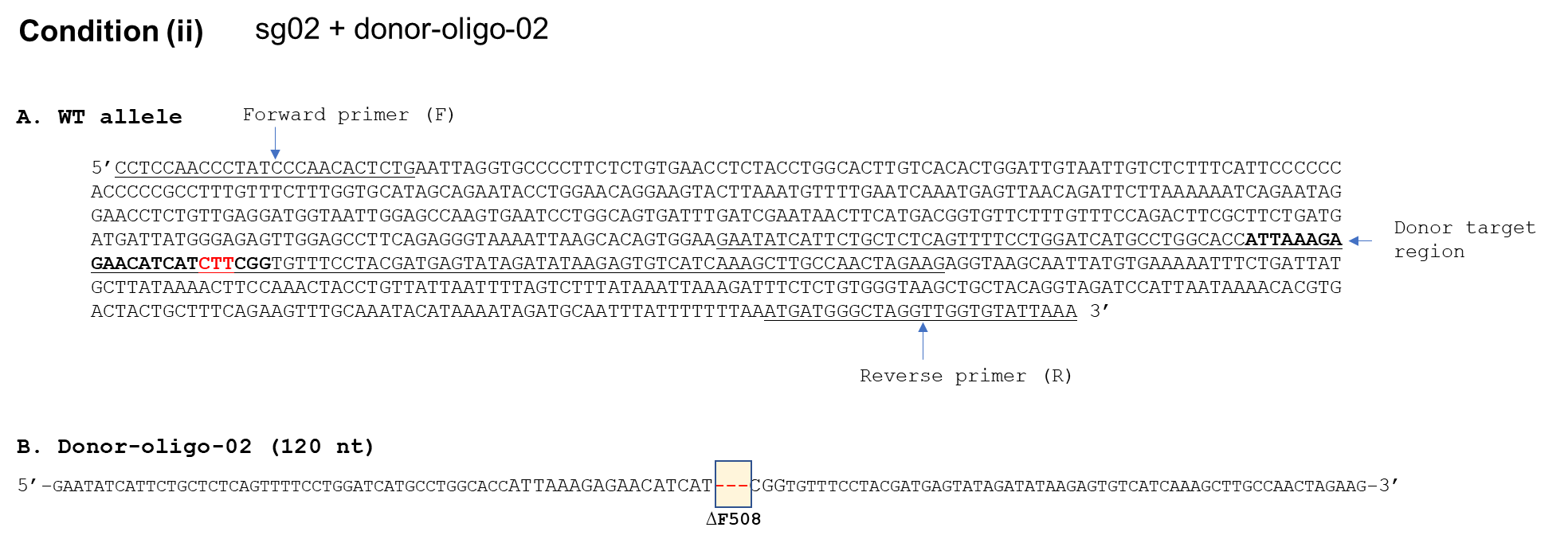
**

**Supplementary Figure 3.** Illustration of Condition (ii). (A) Wild type (WT) allele sequence. Underlined sequences refer to the Forward primer sequence, Reverse primer sequence and the donor target sequence. Bold letters refer to the gRNA sequence. Red letters indicate the F508 locus. (B) Donor-oligo-02 sequence. Red letters indicate mutated nucleotides. Box: dF508 locus.

**
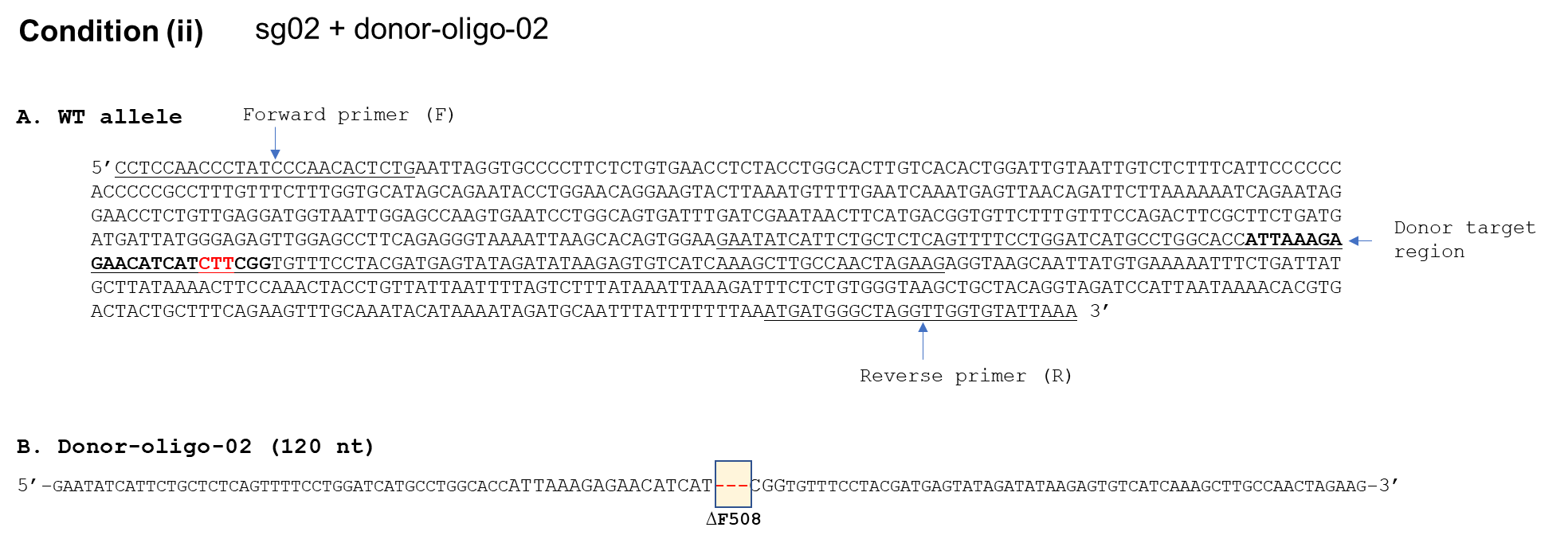
**

**Supplementary Figure 4.** Illustration of Condition (iii). (A) Illustration of the ds-donor-01 design. (B) Sequence of ds-donor-01 proximate to the target locus. Red dots point to silently mutated nucleotides. (C) Full sequence of ds-donor-01. Red letters indicate mutated nucleotides. Box: dF508 locus.

**
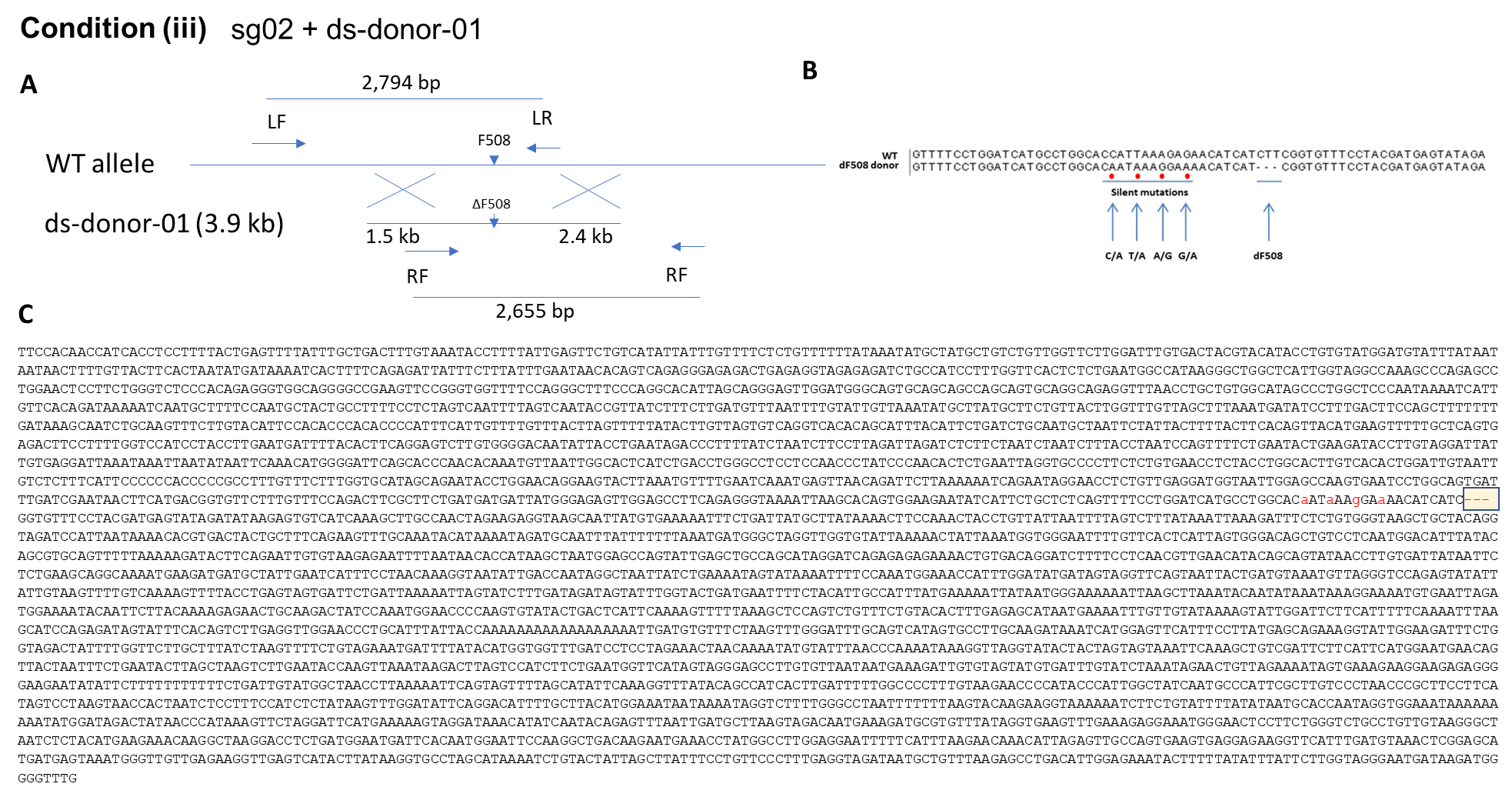
**

**Supplementary Figure 5.** Example sequence analysis output by the ICE online tool of one F1 generation ∆F508/WT heterozygous animal.

**
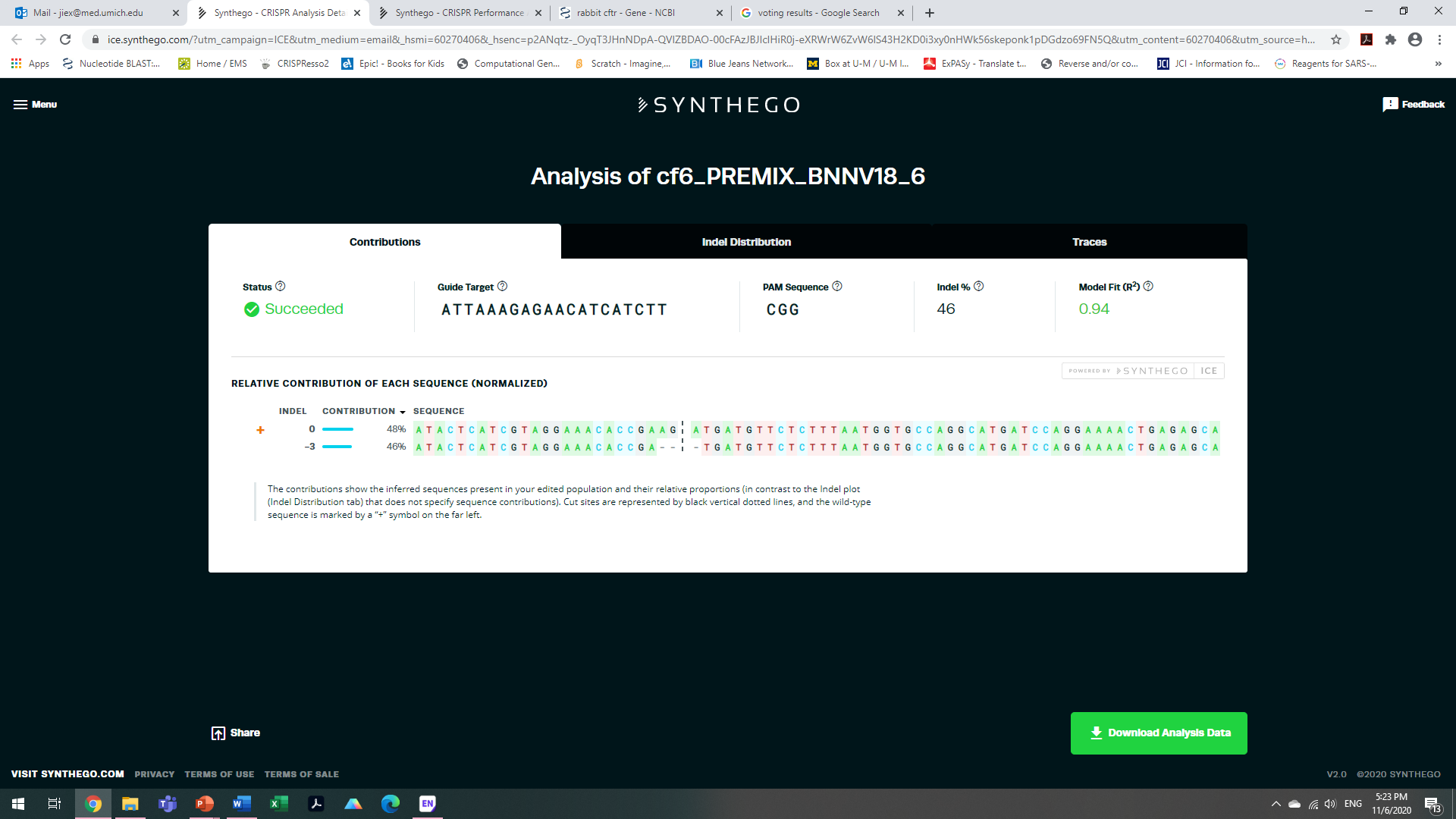
**

**Supplementary Figure 6.** T7E1 assay on potential off-target loci in founder and F1 generation ∆F508 rabbits. 161: ∆F508 founder. 254, 255, 263, 264 and 265: F1 generation ∆F508/WT animals. WT: a wild-type animal. Arrows in each gel: top arrow indicates unedited band size; two bottom bands indicate predicted indel bands. No predicated indel bands were detected in all loci. KALRN: kalirin RhoGEF kinase (NCBI GeneID: 108175385); MED13: Mediator Complex Subunit 13 (NCBI GeneID: 100337925); PKHD1: PKHD1 Ciliary IPT Domain Containing Fibrocystin/Polyductin (NCBI GeneID: 100352414); CANX: calnexin (NCBI GeneID: 100341645). TEX2: testis expressed 2 (NCBI Gene ID: 100350779). NCKAP5: NCK associated protein 5 (NCBI Gene ID: 100349145). RAPH1: Ras association (RalGDS/AF-6) and pleckstrin homology domains 1 (NCBI GeneID: 100351493). TTK: TTK protein kinase (NCBI: GeneID: 100359213).

**
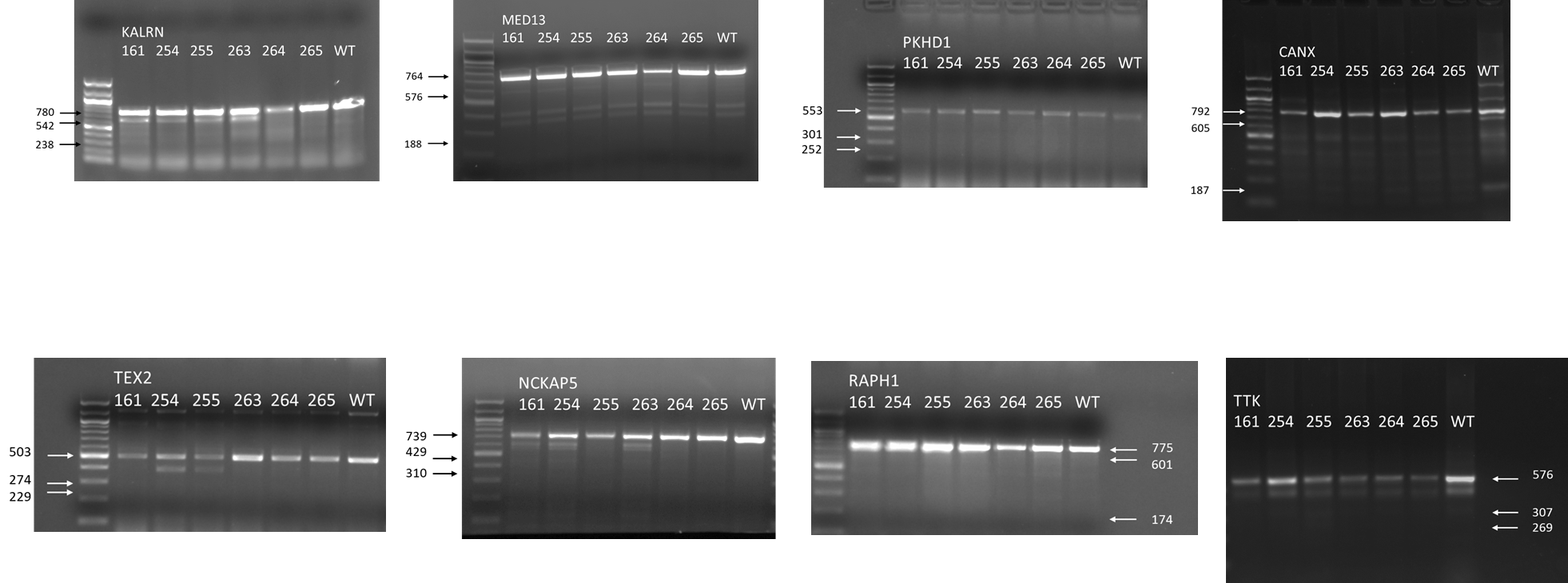
**

**Supplementary Figure 7.** Gross anatomy of CF and WT rabbit intestine. Left: intestinal obstruction of the dF/KO rabbit is indicated by the red arrowhead. Note the absence of feces in the colon. Right: intestine of a healthy WT rabbit. Blue arrow points to a feces pellet.

**
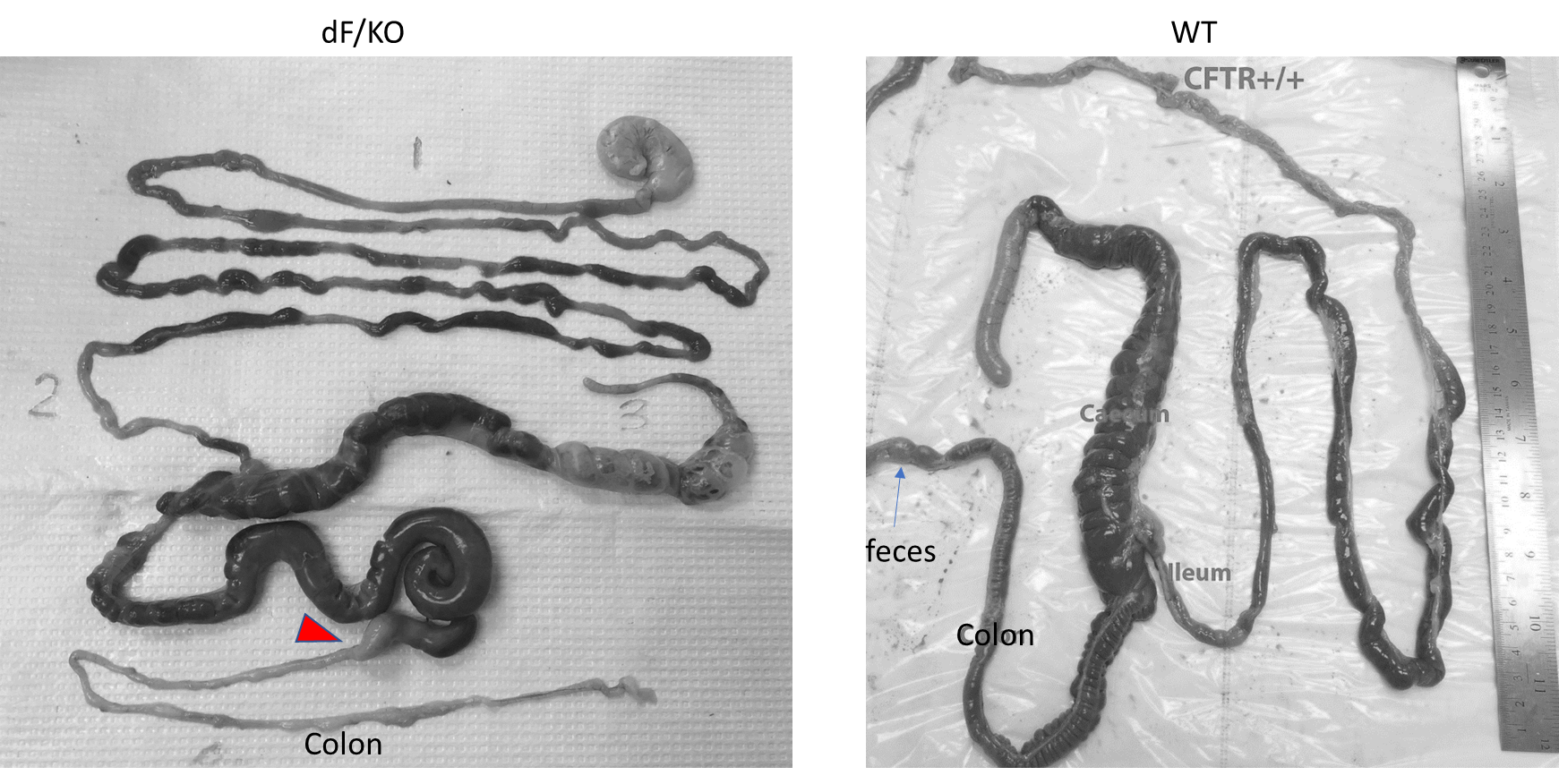
**

**Supplementary Table 1. Top potential off-target sequences**

| # | **Genomic location** | **Locus details** | **Sequence** | **Indel** |
| --- | --- | --- | --- | --- |
|  | sg02-dF |  | ATTAAAGAGAACATCATCTT CGG |  |
| 1 | Chr14: 94575891-94575913 | exon:KALRN | AATAAAGAGCACATCATCTT TGG | No |
| 2 | Chr19: 28197223-28197245 | intron:MED13 | ATTATAAGGCACATCATCTT TGG | No |
| 3 | Chr12: 41453991-41454013 | intron:PKHD1 | ATATCTGAGAACATCATCTT TGG | No |
| 4 | GL018730: 1223477-1223499 | intron:CANX | CATATAGAGAAAATCATCTT TGG | No |
| 5 | Chr19: 49056097-49056119 | intron:TEX2 | AATGAAGAAAAAATCATCTT TGG | No |
| 6 | Chr7: 69908973-69908995 | intron:NCKAP5 | GTGAAAGAGAGTATCATCTT GGG | No |
| 7 | Chr7: 144100036-144100058 | intron:RAPH1 | GTTAGAGAGGACATTATCTT GGG | No |
| 8 | Chr12: 66970989-66971011 | intron:TTK | TTTAAAGTGAAAATCATCTC TGG | No |

**Supplementary Table 2.** Primers for PCR amplification of potential off-target sites.

| **Primer name** | **Sequence** | **Gene/locus** |
| --- | --- | --- |
| KALRN off-F | TGCAGAGAACTGGAGAGGGA | sg02-dF OT1 |
| KALRN off-R | TGAAAAGCCAGCCAGAGGAA |  |
| MED13 off-F | AGGGTGAGGACTAAGTGGGG | sg02-dF OT2 |
| MED13 off-R | GCTTTGTGGTCATTTGCTGC |  |
| PKHD1 off-F | TGCATGCAGACATCCAATTT | sg02-dF OT3 |
| PKHD1 off-R | GCCATGGCTGAGAAAGAACT |  |
| CANX off-F | TGAAAGCAACATCTGGGTGC | sg02-dF OT4 |
| CANX off-R | TCACATCTGGTTGAGGAGCA |  |
| TEX2 off-F | CTGCTAGTTCGTTCCCCAAA | sg02-dF OT5 |
| TEX2 off-R | CTGGCCTCAAGGTTTACCAA |  |
| NCKAP5 off-F | GGGACTCTGAAGCTGTAGTGG | sg02-dF OT6 |
| NCKAP5 off-R | CAGGCTCCTGTCTAACTGCC |  |
| RAPH1 off-F | GGCCAGTTTCACCAGGGATT | sg02-dF OT7 |
| RAPH1 off-R | CCAACCCAATTCGGAGCAGT |  |
| TTK off-F | TTTTGAATTGCACATAAGAGGTTT | sg02-dF OT8 |
| TTK off-R | TGCATCCTAAGGACCAAACA |  |
